# Head Orientation Modulates Vestibular Cerebellar Evoked Potentials (VsCEPs) and Reflexes Produced by Impulsive Mastoid and Midline Skull Stimulation

**DOI:** 10.1007/s12311-023-01587-5

**Published:** 2023-07-19

**Authors:** Sendhil Govender, Daniel Hochstrasser, Neil PM Todd, James G Colebatch

**Affiliations:** 1 School of Clinical Medicine, Randwick Clinical Campus, University of New South Wales, Sydney, NSW 2052 Australia; 2 Neuroscience Research Australia, University of New South Wales, Randwick, Sydney, NSW 2052 Australia; 3 MARCS Institute for Brain, Behaviour and Development, Western Sydney University, Westmead, Sydney, NSW 2145 Australia; 4 Institute of Neurological Sciences, Prince of Wales Hospital, Randwick, Sydney, NSW 2031 Australia

**Keywords:** VEMP, VsCEP, Postural, Vestibular reflexes, Cerebellum

## Abstract

The cerebellum plays a critical role in the modulation of vestibular reflexes, dependent on input from proprioceptive afferents. The mechanism of this cerebellar control is not well understood. In a sample of 11 healthy human subjects, we investigated the effects of head orientation on ocular, cervical, postural and cerebellar short latency potentials evoked by impulsive stimuli applied at both mastoids and midline skull sites. Subjects were instructed to lean backwards with the head positioned straight ahead or held rotated in different degrees of yaw towards the right and left sides. Impulsive mastoid stimulation, a potent method of utricular stimulation, produced localised vestibular cerebellar evoked potentials (VsCEPs: P12-N17) which were strongly modulated by head orientation. The response was larger on the side opposite to the direction of head rotation and with stimulation on the side of rotation. In contrast, ocular VEMPs (oVEMPs: n10-p16) were present but showed little change with head posture, while cervical VEMPs (cVEMPs: p15-n23) were larger with the head held rotated away from the side of the recording. Postural effects with lateral vestibular stimulation were strongly modulated by head rotation, with more powerful effects occurring bilaterally with stimulation on the side of rotation. The duration of the postural EMG changes was similar to the post-excitation inhibition of the electrocerebellogram (ECeG), consistent with cerebellar participation. We conclude that head rotation selectively affects evoked vestibular reflexes towards different targets, consistent with their physiological roles. Changes in VsCEPs may contribute to the modulation of postural reflexes by the cerebellum.

## Introduction

Orientation of the head and thus neck afferents is critical to interpreting the significance of vestibular activation, particularly for any postural significance. A lateral acceleration of the head may signify a lateral trunk acceleration if the head is directed forwards, but it could also signal an antero-posterior acceleration if the head is directed to one side. Nashner and Wolfson [1], using electrical (“galvanic”) stimulation of the vestibular apparatus, showed that the effect of vestibular activation on evoked postural reflexes was profoundly altered by head orientation. With a given stimulus polarity, head rotation from one side to the other reversed the EMG effects in the leg muscles. They pointed out that such a transformation would be expected to occur when transferring from one reference frame (the head) to another, i.e. the trunk. These observations have been repeatedly confirmed [2, 3]. While a variety of receptors can contribute to proprioception [4], it is known that neck muscles contain a high number of muscle spindle afferents [5–7]. These spindle afferents project to the central cervical nucleus which in turn projects monosynaptically to the contralateral vestibular nucleus [8]. The cerebellum appears to be critical for this process to occur [9, 10].

Impulsive stimulation applied to the mastoid has been shown to be an effective method of exciting vestibular receptors, particularly the utricle [11–13]. We have also reported that these stimuli evoke short-latency potentials recorded by electrodes over the cerebellum [14], responses that we have termed “vestibular cerebellar evoked potentials” or VsCEPs. We have proposed that these arise from the Purkinje neurones and represent climbing fibre responses (CFRs), based upon their polarity, lateralisation, amplitude and the post-discharge pause in the electrocerebellogram (ECeG) [14, 15]. Source analysis has confirmed that the generating sites lie within the cerebellum [16]. In a recent study, we found that VsCEPs showed only limited changes in response to body posture [17]. Others have also reported recording independent cerebellar activity, e.g. [18].

The present study was designed to investigate the modulation of vestibular input to the cerebellum, as well as other targets, in response to changes in head posture. The effects of a series of different amounts of head rotations in yaw were examined for both lateralised (mastoid) and midline impulsive stimuli. Head posture was found to have a profound effect on the amplitude of some of the reflexes as well as on VsCEPs.

## Materials and Methods

Eleven normal subjects (42 ± 12 years; 8 male, 3 female) were recruited from staff at the Prince of Wales Hospital. All subjects denied any prior history of vestibular, hearing or neurological impairment. Informed consent was obtained in accordance with the 1964 Declaration of Helsinki, and the study was approved by the local ethics committee (South Eastern Sydney Local Health District Human Research Ethics Committee).

### Impulsive Acceleration Stimuli

The stimulus waveform consisted of impulsive accelerations (a 3rd-order gamma waveform with a 4 ms rise time) generated using a laboratory interface (CED Power1401, Cambridge Electronic Design, Cambridge, UK), a power amplifier (model 2718, Brűel & Kjær, Denmark) and customised software. The stimulus was delivered using a hand-held mini-shaker device (model 4810, Brüel & Kjaer P/L, Denmark) with an attached cylindrical perspex rod (diameter: 2.5 cm, length: 9.2 cm). Impulsive accelerations were applied separately to both the left and right mastoid processes, the nasion (Nz) and the occiput (Oz), with a positive displacement polarity (i.e. movement of the rod towards the subject) and at a fixed intensity of 20 V peak (~14 N peak force level (FL)). The impulsive stimuli were presented for each head position at a rate of ~2 Hz using a total of 100 repetitions.

### Electrocerebellogram (ECeG), Extraocular, SCM and Leg EMG Recordings

ECeG was recorded using a 10-10 cerebellar-extended cap (EASYCAP GmbH, Germany) with a subset of electrodes chosen based on our previous studies (Govender et al. [14, 17]). Five ECeG recording locations were used and consisted of a single row over the posterior of the scalp, at Iz and on either side (PO9, I1, Iz, I2 and PO10) with 10% separation (see Fig. 1B in Govender et al. [17]). A ground electrode was positioned at Cz and a reference electrode at AFz. ECeG signals were amplified (20,000×) and filtered (0.5 Hz to 3 kHz) using two D360 amplifiers (Digitimer Ltd., Welwyn Garden City, UK). Extraocular EMG recordings consisted of recording electrodes beneath the eyes on the orbital rim and reference electrodes 2 cm below them (EO1 and EO2). Further EMG recordings were made bilaterally from the sternocleidomastoid (SCM) and lower limb (soleus and tibialis anterior (TA)) muscles using surface adhesive electrodes (Cleartrace 1700-030, CONMED Corp., USA). For the SCM recordings, active electrodes were positioned just above halfway up the belly of the SCM and referred to an electrode over the ipsilateral sternal tendon. For the leg muscles, recordings were made 1–2 cm above the musculotendinous junction for the soleus and 1–2 cm lateral to the tibia for the TA, with reference electrodes 2 cm below the active electrodes. Peaks were named by their latencies preceded by i- (ipsilateral) or c- (contralateral) in relation to the mastoid stimulated. A ground electrode was placed on the midpoint of the right lower leg. EMG signals were amplified (2500×) and filtered (8 Hz to 1.6 kHz) using AA6 Mk III amplifiers (Medelec Ltd., Old Woking, Surrey, UK).

**Fig. 1:**
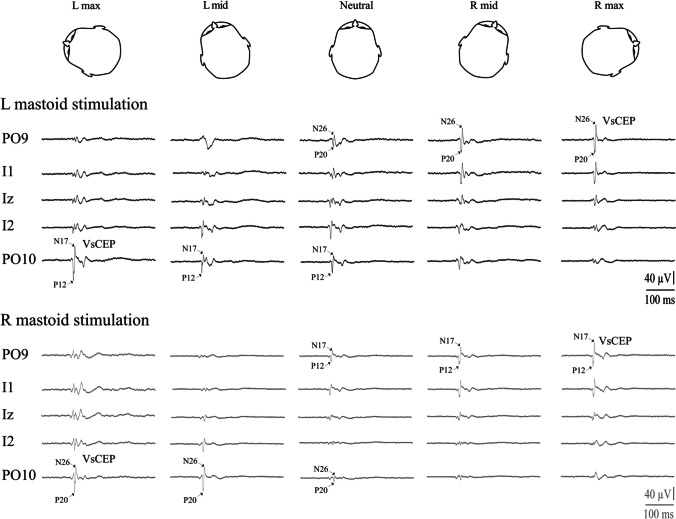
Single-subject data showing changes in VsCEP responses to the left (black traces, upper) and right (grey traces, lower) mastoid stimulation. The effect of head position was qualitatively the same for both mastoid sites. Stimulation ipsilateral to the direction of head rotation (e.g. left, upper left trace) evoked a large VsCEP with a short latency in the electrode overlying the opposite cerebellar hemisphere (P12-N17; PO10). Stimulation over the opposite (right) mastoid (lower set of traces), with the head rotated to the left, evoked a large but later response in the same electrode. The opposite occurred with head rotation to the right; here, right mastoid stimulation evoked a large short-latency response in PO9, with left mastoid stimulation evoking a later response

Recordings were made using Signal software (version 6.02, Cambridge Electronic Design, Cambridge, UK) and a Micro1401 (Cambridge Electronic Design, Cambridge, UK), with sampling at 10 kHz from 100 ms prior to the stimulus onset to 300 ms after it. Leg muscle EMG was rectified and averaged offline using MATLAB software (R2007b, Mathworks, Natick, CA).

### Experimental Procedure

Subjects stood upright and were asked to lean backwards during the recordings to increase tonic activity in the TAs. Five head positions in yaw were tested, with each mastoid stimulated separately. When stimulating the mastoid, subjects were recorded while holding their heads rotated as far as comfortably possible to either the right or left sides (maximum right and left head positions; R and L max), to approximately 45° to either the right or left (R and L mid), and while looking forwards with the head straight (neutral head position). A stimulation was also carried out using Oz and Nz as stimulus sites using three head positions (head held maximally rotated to the right and left sides and in the neutral position). In total, sixteen conditions were recorded for each subject, and the order of presentation was randomized between subjects. All responses were recorded simultaneously.

### Data Analysis

Peak-to-peak biphasic peaks were measured from the ipsilateral and contralateral sides with respect to the stimulated mastoid. For ocular vestibular evoked myogenic potentials (oVEMPs), the c-n12/p17 peaks and i-n15/p21 initial peaks were measured from the extraocular muscles [19], as well as the later peaks (second and third negativities and second positivity). For cervical vestibular evoked myogenic potentials (cVEMPs), the i-p15/n23 and c-n15/p20 peaks were measured for the ipsilateral and contralateral SCM respectively [20]). For SCM responses, raw amplitudes were adjusted for differences in background activity during head rotations and expressed as a corrected ratio [21]. Both raw and corrected amplitudes were used for analyses. Cerebellar-evoked responses were measured for the c-P12/N17 and i-P20/N26 peaks [14]. VsCEPs were identified by their latency, localisation and short interpeak intervals (mean 4.4 ms). Short-latency (SL) leg muscle responses were rectified and averaged, with amplitudes normalised to baseline levels. Six of the eleven subjects had recordings with clear VsCEPs that were not contaminated by high-frequency noise or stimulus artefacts and were used to analyse changes in power. Grand mean scaleograms (time-frequency images) were produced using the continuous wavelet transform (CWT) in MATLAB (R2019b, see Todd et al. [22]). Scaleograms were then further split into several bands, and the very high-frequency band (VHF: 160-320 Hz) was selected for detailed analysis. RMS (root mean square) averages with VHF filtering (160-320 Hz) were also constructed offline using MATLAB software. Both the tonic (baseline) activity levels and the post-response period of inhibition were measured from the VHF RMS averages, the latter quantified as the percentage decrease from baseline levels.

For recordings over the posterior fossa, a repeated measures ANOVA was performed using the site of stimulation (right and left mastoid) electrode (PO9, I1, Iz, I2 and PO10) and head position (max left, mid left, neutral, mid right and max right) as factors (IBM SPSS Statistics for Windows, Version 26.0. Armonk, NY: IBM Corp.). ANOVAs for extraocular responses, SCM-evoked responses and baseline TA levels were performed using side (ipsilateral and contralateral to the stimulated mastoid) and head position as factors. Peak latencies were analysed separately using peaks and head position as factors. For midline stimulation, electrode (in turn, oVEMP: EO1/2, VsCEP: PO9/10, cVEMP: R/L SCM and lower limb: R/L TA) and head position (max left, neutral and max right) were used as factors. The Greenhouse-Geisser correction was used to correct for the violation of the assumption of sphericity. Bonferroni’s correction was applied for post hoc *t*-tests. Pearson’s correlation was used to compare the VsCEP and SL amplitudes. Values are given as mean ± SD in tables and mean ± SEM in graphs.

## Results

### Modulation of VsCEPs and Spectral Power with Changing Head Yaw Position

**Fig. 2:**
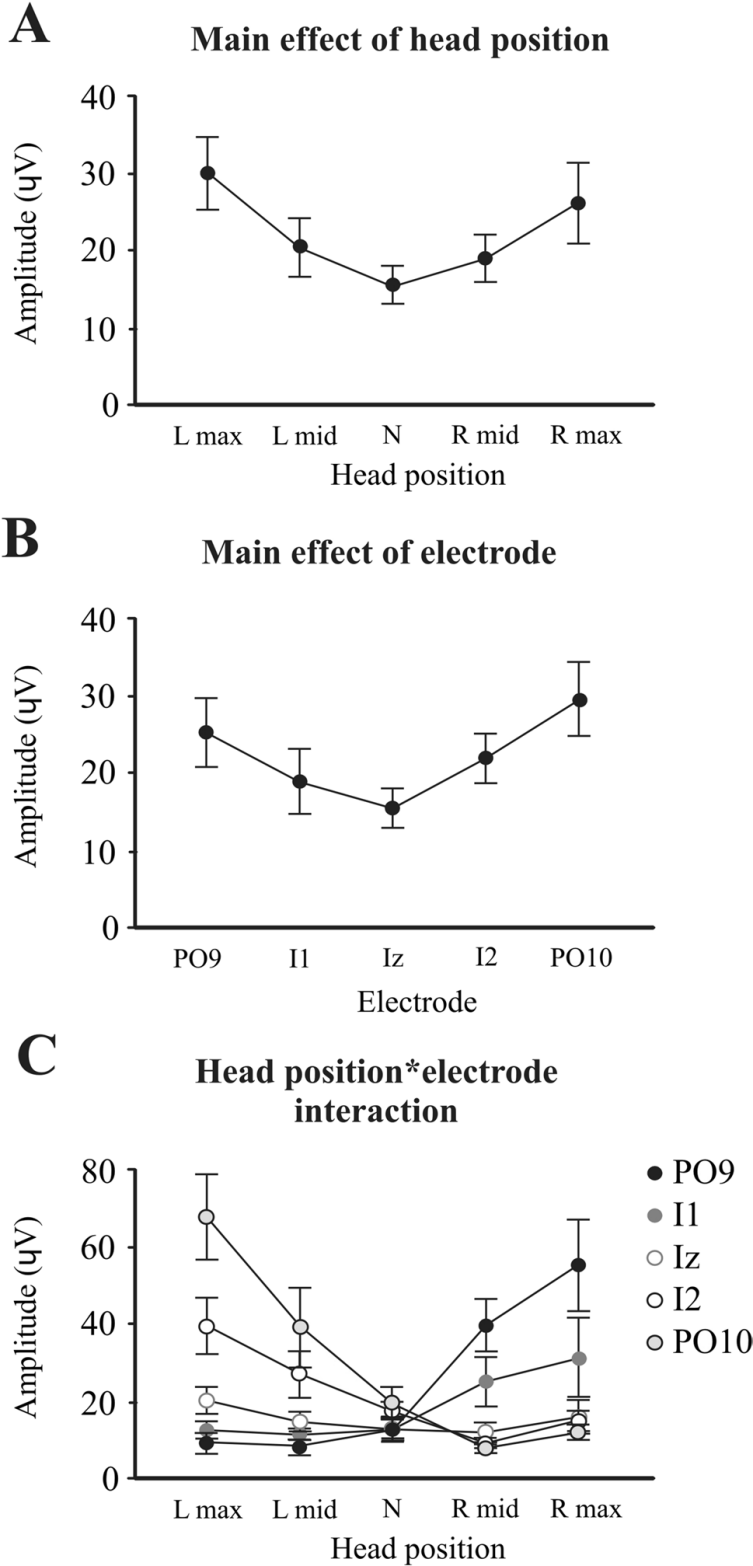
ANOVA main and interaction effects. Main effect of head position (**A**) shows larger responses with maximal head rotation to the left and right sides. The main effect of the electrode (**B**) demonstrates larger responses for the more lateral PO9 and PO10 electrodes. The head position and electrode interaction for VsCEP amplitudes (**C**) show responses increasing on the side opposite to the direction of head rotation, more so for the PO9 and PO10 electrodes. R, right; L, left; N, neutral

Impulsive accelerations applied to both the right and left mastoids produced short latency potentials occurring contralateral (c-P12/N17) and ipsilateral (i-P20/N26) to the stimulated mastoid. Overall, VsCEPs were strongly modulated by changes in head position (Fig. 1 and Table 1). Individually, in the head neutral position, 8 of the 11 subjects had VsCEPs in one (*n* = 2) or both directions of stimulation (*n* = 6), with interpeak amplitudes up to 101 μV (mean 22 μV). With the head rotated, all but one subject had contralateral VsCEPs in response to stimulation of the mastoid ipsilateral to the direction of head rotation (mean amplitude 65.3 μV). One subject had a VsCEP only with left mastoid stimulation, with the head rotated to the left. On average, maximal rotation of the head to either the right or left produced larger evoked responses (Fig. 2A; *F*_(2.4,42.2)_ = 8.9, *P* = 0.001), with the amplitudes recorded from the most lateral electrode locations (PO9 and PO10) being the largest (Fig. 2B; *F*_(2.6,25.7)_ = 9.9, *P* < 0.001). As the degree of head rotation changed from one side to the other, the evoked responses increased on the side opposite to the direction of head rotation for both sides of stimulation (Fig. 2C; *F*_(1.6,16.2)_ = 14.5, *P* < 0.001). For the PO10 electrode, a maximal head rotation to the left produced significantly larger amplitudes than at mid-leftward head rotation (*t*_(10)_ = 3.7, *P* = 0.006), and the amplitudes also tended to decrease as the head rotated from mid-leftward head to mid-rightward (*t*_(10)_ = 2.4 and 2.6, *P* = 0.045 and 0.028, not significant after Bonferroni’s correction). For the PO9 electrode, a maximal rotation to the right tended to produce larger amplitudes than mid-rightward head rotation (*t*_(10)_ = 2.3, *P* = 0.052), whereas the mid-rightward head rotation produced significantly larger amplitudes than the neutral position (*t*_(10)_ = 4.2, *P* = 0.003).

**Table 1:** Mean initial peak-to-peak VsCEP amplitudes across the differing head positions and posterior fossa electrodes

*Head position*					
	*L max*	*L mid*	*Neutral*	*R mid*	*R max*
L mastoid stimulation					
PO9	8.0 (7.3)	7.6 (6.9)	6.8 (5.5)	30.0 (22.9)	51.1 (46.5)
I1	10.2 (6.3)	10.4 (4.6)	8.9 (4.0)	18.7 (18.1)	29.1 (37.4)
Iz	18.3 (12.2)	14.9 (9.0)	13.2 (8.1)	8.8 (6.9)	16.0 (16.3)
I2	35.7 (21.8)	25.5 (18.9)	22.1 (15.0)	9.0 (4.3)	18.5 (11.8)
PO10	63.8 (35.3)	37.0 (30.3)	25.1 (25.0)	7.8 (4.5)	12.4 (8.0)
R mastoid stimulation					
PO9	11.5 (11.0)	9.7 (8.4)	18.9 (18.1)	49.4 (27.2)	59.3 (33.9)
I1	15.7 (11.1)	13.2 (8.3)	17.8 (17.1)	31.8 (28.0)	33.9 (32.1)
Iz	22.9 (14.7)	15.2 (10.6)	13.3 (8.5)	16.0 (11.7)	16.7 (12.1)
I2	43.6 (33.9)	28.9 (27.9)	13.5 (7.4)	10.2 (5.9)	12.1 (8.7)
PO10	71.2 (45.9)	41.8 (42.5)	14.7 (8.3)	8.2 (5.1)	12.5(10.1)

Mean (SD), *R*, right; *L*, left

**Fig. 3:**
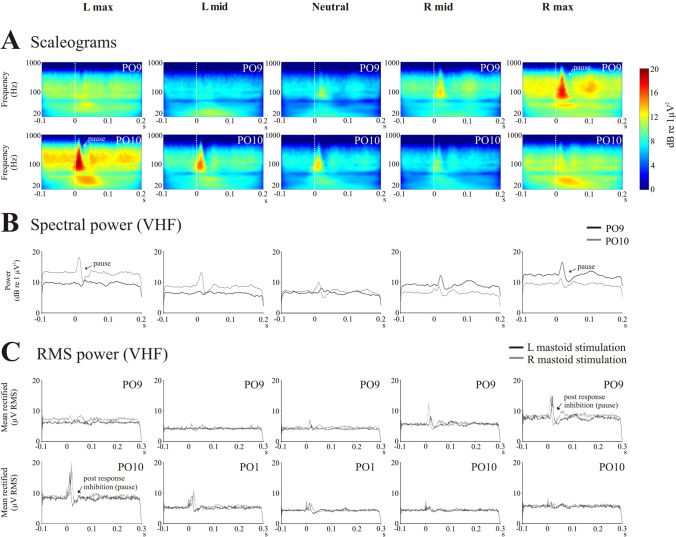
Scaleograms (**A**), spectral power (**B**) and RMS power (**C**) across the differing head positions for the PO9 and PO10 electrodes (*n* = 6). Scaleograms (top two rows) show the characteristic pausing in ECeG following the evoked response, and this became more prominent on the side opposite to the direction of maximal head rotation. This can also be seen in the extracted VHF power (middle row). Scaleograms and spectral power are shown for left mastoid stimulation. RMS averages showed the same pattern of modulation, with the post-response inhibition (pause) becoming larger on the side opposite to maximal head rotation (bottom two rows). The difference in latency for the two sides of stimulation is also evident in the RMS averages. Note some changes in tonic power occurring with head rotation. s, seconds

**Fig. 4:**
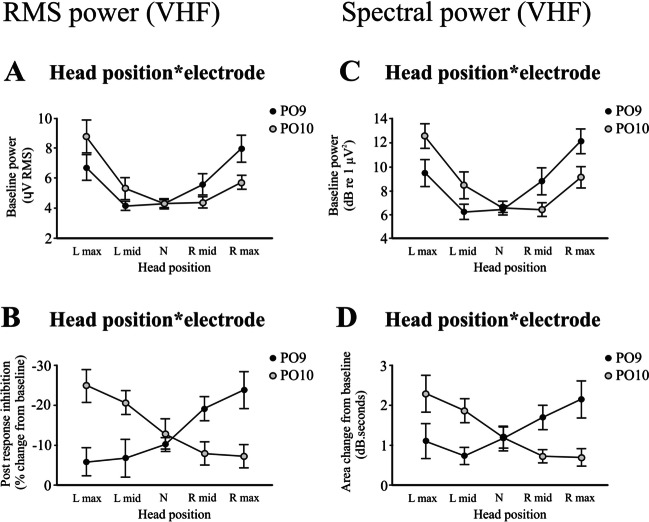
Head position by electrode interaction effects for the cerebellar (PO9 and PO10) electrodes obtained from RMS (left column) and spectral (right column) power in the VHF band (*n* = 6). The baseline RMS power (**A**) increased bilaterally with maximal head rotation but was larger on the opposite side (PO9 for right maximal rotation and PO10 for left maximal rotation). The post-response inhibition (**B**) also increased and became larger on the side opposite to the direction of maximal head rotation. Analysis of spectral power produced similar results (**C**, **D**)

Scaleograms (Fig. 3A) constructed from the PO9 and PO10 electrodes showed changes in power across the differing head positions. Spectral power demonstrated modulation in the higher frequency bands, and we chose the VHF band for subsequent analyses using both extracted spectral power (Fig. 3B) and RMS averaging (Fig. 3C). RMS power confirmed the post-response inhibition previously reported [14], and changes in tonic RMS power with head position generally mirrored that of the evoked response. The baseline RMS power increased bilaterally with maximal head rotation to either side, but the increase was greater on the side opposite to the direction of rotation (Fig. 4A; *F*_(1.5,7.7)_ = 6.3, *P* = 0.028). Similar to the evoked response, the magnitude of the post-response inhibition increased markedly for the response on the side opposite to the direction of maximal head rotation (mean −6.8 to −24.3%, Table 2 and Fig. 4B; *F*_(1.6,8.1)_ = 17.9, *P* = 0.001). Onset and end latencies of the post-response inhibition were unaffected by head position (onset: *F*_(2.4,12.0)_ = 1.1, *P* = 0.364; end: *F*_(1.8,8.8)_ = 0.2, *P* = 0.797). Analysis of VHF spectral power yielded similar results (Fig. 4C, D).

**Table 2:** Baseline RMS and post-response inhibition amplitudes and latencies for the differing head positions

*Head position*					
	*L max*	*L mid*	*Neutral*	*R mid*	*R max*
L mastoid stimulation					
Baseline (μV RMS)					
PO9	6.1 (1.7)	4.1 (0.9)	4.1 (0.8)	5.2 (2.0)	7.6 (1.9)
PO10	8.3 (2.1)	5.2 (1.9)	4.3 (0.9)	4.3 (1.0)	5.6 (1.1)
Inhibition amplitude (% change from baseline)					
PO9	4.2 (6.5)	0.3 (15.5)	−7.2 (4.0)	−16.3 (8.5)	−18.2 (11.5)
PO10	−23.5 (11.2)	−21.3 (8.6)	−15.7 (13.9)	−9.3 (11.3)	−9.5 (11.0)
Inhibition onset (ms)					
PO9	28.9 (1.0)	29.6 (2.7)	31.9 (9.2)	29.8 (7.9)	26.7 (4.1)
PO10	18.5 (2.2)	19.3 (2.1)	19.3 (2.8)	21.4 (2.1)	19.4 (1.4)
Inhibition end (ms)					
PO9	58.1 (3.6)	58.2 (6.2)	61.0 (20.4)	62.6 (13.4)	55.7 (17.0)
PO10	42.4 (12.9)	43.1 (9.8)	47.7 (9.2)	44.3 (4.9)	40.4 (3.4)
R mastoid stimulation					
Baseline (μV RMS)					
PO9	7.3 (2.7)	4.2 (0.7)	4.5 (1.0)	5.7 (1.6)	8.3 (2.6)
PO10	9.3 (3.5)	5.4 (1.7)	4.4 (0.8)	4.5 1.2()	5.9 (1.4)
Inhibition amplitude (% change from baseline)					
PO9	−16.3 (14.5)	−14.3 (12.4)	−13.2 (5.9)	−22.1 (8.3)	−29.6 (11.2)
PO10	−26.3 (11.5)	−20.2 (6.8)	−9.9 (10.4)	−7.0 (10.2)	−5.7 (16.0)
Inhibition onset (ms)					
PO9	20.5 (2.1)	20.1 (1.7)	20.3 (2.3)	20.7 (3.7)	18.1 (2.3)
PO10	26.3 (3.5)	27.9 (3.9)	31.9 (4.0)	30.1 (6.1)	31.7 (6.2)
Inhibition end (ms)					
PO9	44.2 (8.1)	44.4 (7.3)	39.8 (3.2)	48.9 (8.8)	43.0 (10.9)
PO10	52.3 (13.6)	55.8 (14.8)	56.1 (8.5)	54.4 (9.8)	51.1 (8.1)

Mean (SD), *R*, right; *L*, left

### Comparison of Extraocular, Cerebellar, SCM and Lower Limb Evoked Responses with Changing Head Position

**Fig. 5:**
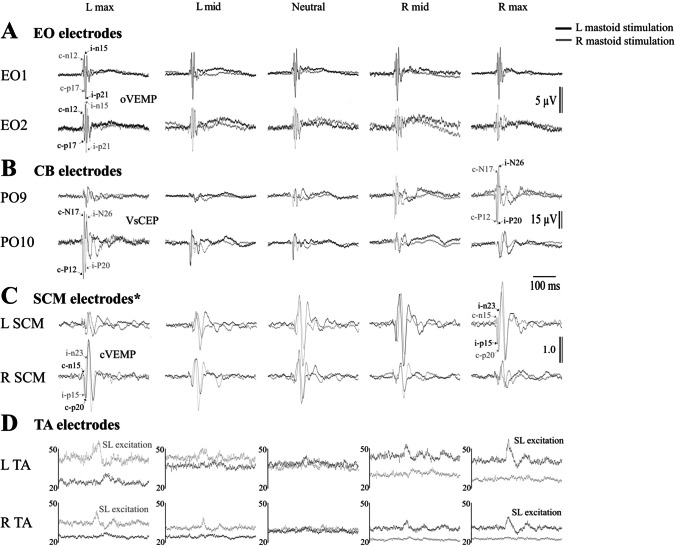
Grand mean responses (*n* = 11) from the extraocular, cerebellar, sternocleidomastoid and tibialis anterior (TA) electrodes. Traces are shown for left (black traces) and right (grey traces) mastoid stimulation. Cerebellar responses (CEPs) are largest for both directions of perturbation from the electrode opposite to the direction of head rotation. The oVEMPs show little change in response to head rotation, while the cVEMP in SCM shows enhancement by head rotation to the opposite side, after allowing for tonic changes in activation. Postural reflexes shown for TA demonstrate clear changes with well-formed responses with head rotation to the side opposite stimulation and attenuation of the effect with rotation back to the midline and beyond, but with corresponding changes in tonic activity. *Responses from the SCM have been divided by pre-stimulus SCM activation levels and therefore reflect the corrected ratio

Figure 5 illustrates the grand mean evoked responses for the ocular (oVEMP), cerebellar (VsCEP recorded from PO9 and PO10), SCM (cVEMP) and tibialis anterior (TA) electrode locations. Given the similarity in the responses for both mastoid sites, we recoded the sides ipsilateral and contralateral to the stimulated mastoid for analyses (Table 3). Head position effects differed fundamentally between electrode locations.

**Table 3:** oVEMP, cVEMP and postural evoked responses across the differing head positions

*Head position*					
	*Max*	*Mid*	*Neutral*	*Mid*	*Max*
	*(towards stimulated mastoid)*			*(away from stimulated mastoid)*	
Extraocular electrodes (oVEMP)					
Amplitude (μV)					
Ipsi (n15/p21)	12.8 (7.0)	12.1 (7.8)	12.7 (8.1)	14.1 (7.6)	16.3 (9.5)
Contra (n12/p17)	9.4 (5.1)	9.4 (4.5)	9.9 (5.5)	11.4 (5.4)	11.5 (5.3)
Latency (ms)					
Ipsi (n15)	16.3 (1.4)	16.3 (1.4)	16.2 (1.5)	15.8 (1.7)	16.0 (1.5)
Ipsi (p21)	22.0 (1.9)	21.5 (2.2)	21.8 (1.6)	21.3 (1.7)	21.6 (1.9)
Contra (n12)	12.9 (2.1)	13.2 (2.9)	12.5 (2.3)	12.3 (2.4)	12.1 (2.0)
Contra (p17)	18.0 (2.1)	18.6 (3.6)	18.4 (3.6)	17.8 (3.4)	18.0 (3.0)
SCM electrodes (cVEMP)					
SCM activation (μV)					
Ipsi	16.4 (6.3)	16.3 (8.1)	19.4 (10.6)	27.5 (11.5)	52.5 (24.4)
Contra	39.4 (21.3)	21.0 (10.7)	16.8 (9.7)	16.2 (8.0)	17.3 (5.9)
Raw amplitude (μV)					
Ipsi (p15/n23)	12.5 (7.1)	9.7 (7.2)	16.6 (13.7)	36.6 (25.1)	76.7 (47.2)
Contra (n15/p20)	55.3 (42.9)	20.6 (18.7)	10.6 (11.5)	8.9 (5.2)	13.2 (10.3)
Corrected ratio					
Ipsi (p15/n23)	0.8 (0.4)	0.6 (0.4)	0.8 (0.4)	1.2 (0.5)	1.4 (0.4)
Contra (n15/p20)	1.2 (0.4)	0.9 (0.5)	0.6 (0.3)	0.6 (0.3)	0.8 (0.6)
Latency (ms)					
Ipsi (p15)	16.1 (1.2)	15.8 (0.8)	15.6 (0.9)	15.9 (0.9)	15.8 (1.5)
Ipsi (n23)	25.8 (2.0)	25.3 (2.1)	25.1 (1.9)	24.9 (1.8)	24.5 (1.6)
Contra (n15)	13.2 (1.1)	13.4 (0.8)	14.5 (0.8)	14.2 (1.0)	14.0 (0.9)
Contra (p20)	21.9 (2.2)	21.3 (1.8)	21.7 (1.8)	20.8 (1.9)	21.0 (1.9)
TA electrodes					
Baseline (μV)					
Ipsi	15.4 (14.9)	17.7 (17.1)	22.3 (20.9)	26.8 (25.7)	31.9 (31.5)
Contra	18.7 (18.3)	19.5 (20.7)	24.3 (23.4)	27.5 (25.9)	28.1 (27.6)
Amplitude (% increase from baseline)					
Ipsi (SL)	-	-	-	15.0 (10.0)	22.0 (14.4)
Contra (SL)	-	-	-	15.3 (8.1)	26.5 (14.0)
Latency (ms)					
Ipsi (SL onset)	-	-	-	51.5 (2.8)	52.8 (3.9)
Ipsi (SL end)	-	-	-	86.4 (7.2)	86.3 (5.3)
Contra (SL onset)	-	-	-	51.5 (2.8)	53.1 (4.0)
Contra (SL end)	-	-	-	85.7 (7.3)	85.7 (5.2)

Mean (SD), *Ipsi*, ipsilateral; *contra*, contralateral to the stimulated mastoid. Values reflect the average of right and left mastoid stimulation

**Fig. 6:**
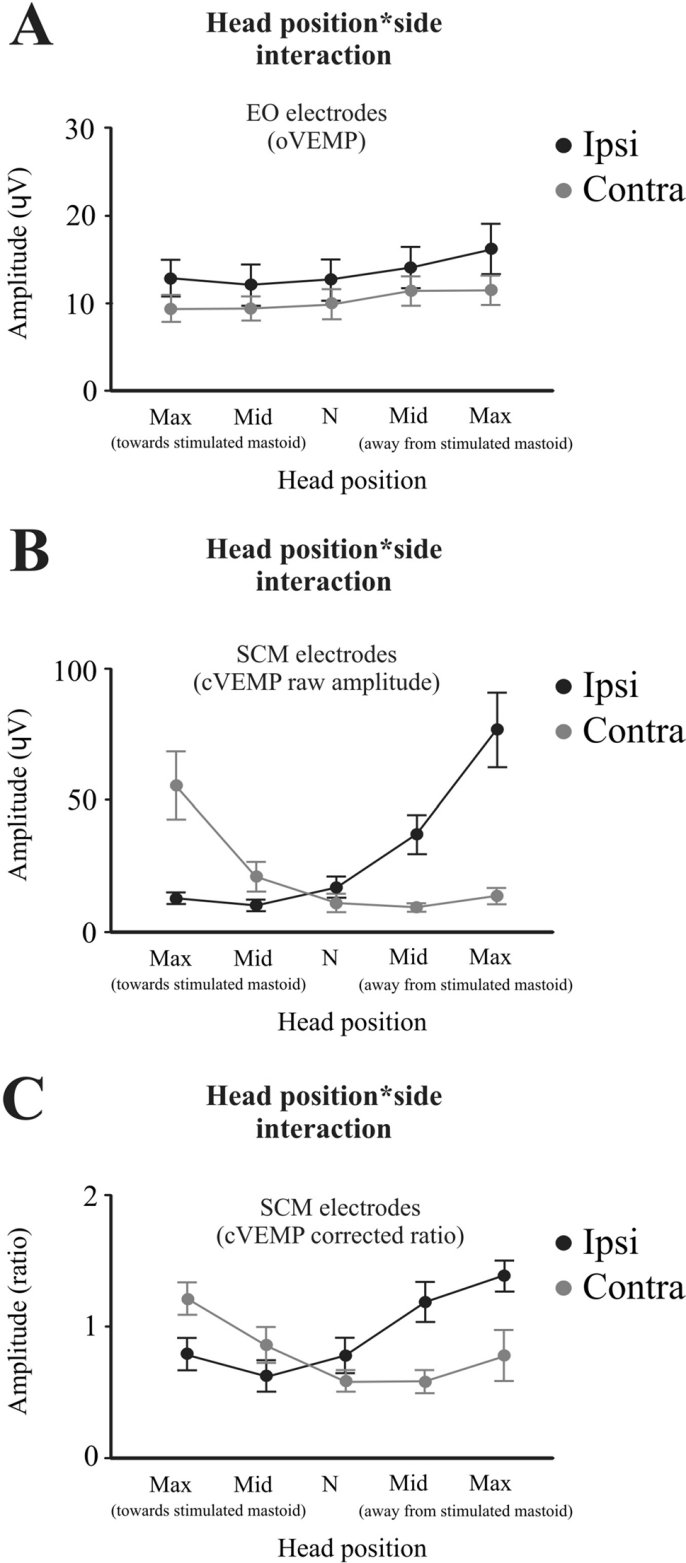
Head position by electrode interaction effects for the extraocular and SCM electrodes. Unlike VsCEP responses, oVEMP amplitude (**A**) showed no significant modulation by head position. For the SCM electrodes, cVEMP raw amplitude was largest for head rotation towards (contra response: n15/p20) and away (ipsi response: p15/n23) from the stimulated mastoid (B) and this was still evident after correcting for the differing levels of activation (C)

For the extraocular electrodes, oVEMP peaks differed between the ipsilateral and contralateral sides of the stimulus. The initial amplitude was slightly larger on the same side as the stimulated mastoid (overall means: 13.6 vs. 10.3 μV; *F*_(1,10)_ = 7.1, *P* = 0.023). In contrast to the VsCEP, initial oVEMP amplitudes on neither side were significantly modulated by head position (Fig. 6A; *F*_(2.5,24.8)_ = 0.9, *P* = 0.425). Amplitudes for the later negative and positive waves (second and third negativities and second positivity) were also not modulated by head position (*F*_(5.9,58.9)_ = 1.2, *P* = 0.312). The latencies of the c-n12, c-p17, i-n15 and i-p21 oVEMP peaks were unaffected by head position (*F*_(3.4,34.8)_ = 1.0, *P* = 0.340).

For the SCM electrodes, the cVEMP amplitude increased on the side opposite to the direction of maximal head rotation (Fig. 6B; raw amplitude: *F*_(1.3,12.9)_ = 16.3, *P* < 0.001), and this effect was present even after accounting for differences in background muscle activation between head positions (Fig. 6C; corrected amplitude: *F*_(1.7,17.3)_ = 8.6, *P* = 0.003). For the response ipsilateral to the stimulus (p15/n23: Table 3), the raw cVEMP amplitude increased 6.13 times (15.7 dB) between the two extremes of head rotation. The background activation increased 3.2 times (10.1 dB), while the head position increased the response by 1.9 times (5.6 dB). The latencies of the i-p15, i-n23, c-n15 and c-p20 cVEMP peaks were unaffected by head position (*F*_(3.7,36.9)_ = 1.6, *P* = 0.176).

**Fig. 7:**
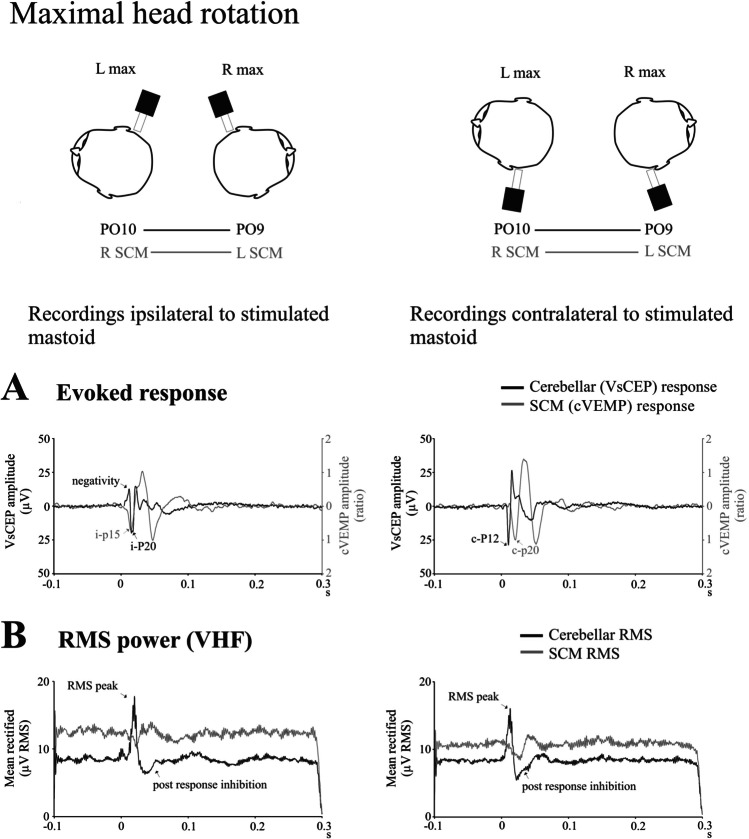
Evoked responses (**A**) and RMS power (**B**) during maximal head rotation (*n* = 6). The schematic illustrates electrode pairs used during maximal head rotation to construct evoked responses and RMS power averages shown in **A** and **B**. The traces compare responses from the cerebellar (black traces) and SCM (grey traces) recording electrodes on the side ipsilateral (left column) and contralateral (right column) to the stimulated mastoid. The responses show different latencies and interpeak intervals. Responses from the SCM were divided by pre-stimulus SCM activation levels and therefore reflect the corrected ratio. In addition, no RMS peak was present for the SCM recordings which just showed inhibition

Given the similarities in the modulation between lateral cerebellar and SCM responses with head rotation and their proximity, we compared the evoked responses and RMS high-frequency power from the two sites during maximal head rotation. In both cases, there were significant differences in the initial latencies between the two responses. For the evoked responses from the side ipsilateral to mastoid stimulation (Fig. 7A, left column), the initial positivity from the SCM occurred earlier than the cerebellar response (i-p15 cVEMP vs. c-P20 VsCEP; 15.2 vs. 17.6 ms; *t*_(11)_ = 3.7, *P* = 0.004). For the side contralateral to the stimulated mastoid (Fig. 7A, right column), the opposite applied—the initial positivity from the cerebellar electrodes occurred earlier than the positivity from the SCM electrodes (c-P12 VsCEP vs. c-p20 cVEMP; 11.1 vs. 21.6 ms; *t*_(11)_ = 15.3, *P* < 0.001). Mean baseline RMS levels were slightly higher from the SCM electrodes, but the overall levels were not significantly different between the two sites for both the ipsilateral (CB vs. SCM; 8.4 vs. 12.5 μV; *t*_(11)_ = 1.8, *P* = 0.096) and contralateral sides (8.3 vs. 10.7 μV; *t*_(11)_ = 1.4, *P* = 0.187). The RMS peak preceding the post-response inhibition was present only in the cerebellar electrodes (Fig. 7B, both columns). The figure also demonstrates a typical feature of the VsCEP in addition to its localisation—its rapid decline and rise, giving much shorter interpeak intervals than for a cVEMP.

For the lower limbs, unlike other recording sites, the evoked responses in TA were similar for the muscles of both the ipsilateral and contralateral legs. In both cases, a response was present in the TA muscles, most clearly when the stimulated mastoid potentially induced a perturbation which posed a threat to stability. For the adopted posture of posterior lean, this meant rightward head rotation with left mastoid stimulation and leftward head rotation with right mastoid stimulation (Fig. 5). Baseline TA levels increased significantly as the head rotated away from the stimulated mastoid (Table 3; *F*_(1.4,13.7)_ = 6.8, *P* = 0.015). With maximal head rotation away from the stimulated mastoid, there was no difference in average SL onset (Table 3, 51.5–53.1 ms, *t*_(10)_ = 1.2, *P* = 0.263) and SL end latencies (85.7–86.4 ms; *t*_(10)_ = 0.9, *P* = 0.350) between the ipsilateral and contralateral TA muscles, whereas SL amplitude was slightly larger for the leg contralateral to the direction of rotation (26.5 vs. 22.0%; *t*_(10)_ = 2.6, *P* = 0.025). The evoked average SL amplitude did not correlate with the corresponding VsCEP amplitude (*r*_(20)_ = 0.08, *P* = 0.739).

### Midline Stimulation

**Fig. 8:**
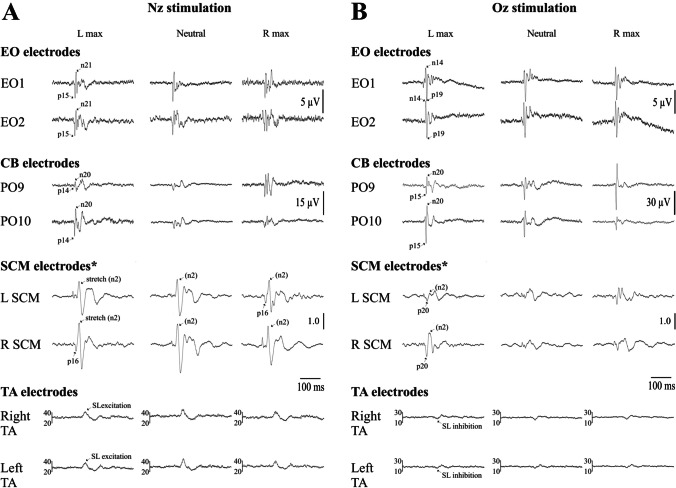
Grand means (*n* = 11) for the extraocular, cerebellar, SCM and lower limb (TA) electrodes following midline stimulation at Nz (**A**) and Oz (**B**). The oVEMPs inverted their initial polarity but did not change in amplitude. CEPs were largest at the electrode contralateral to the direction of head rotation. SCM cVEMPs were contaminated by muscle stretch responses but were larger on the side contralateral to the direction of head rotation. Rectified averages are shown for the TA muscles which, unlike the others, showed the same effects on both sides. There was little effect of head rotation, but the responses were inverted for the two different stimulus sites. *Responses from the SCM were divided by pre-stimulus SCM activation levels and therefore reflect the corrected ratio. Units for the TA electrodes are in μV

Midline stimulation at Nz and Oz produced similar results to that of mastoid stimulation (Fig. 8 and Tables 4 and 5) but were different for postural responses.

**Table 4:** oVEMP, CEP, cVEMP and TA postural evoked responses across the differing head positions to Nz stimulation

	*Head position*		
	*L max*	*Neutral*	*R max*
Extraocular electrodes (oVEMP)			
Amplitude (μV)			
EO1 (p15/n21)	6.9 (3.8)	7.9 (4.2)	7.0 (3.9)
EO2 (p15/n21)	8.0 (4.3)	6.8 (4.4)	6.7 (4.3)
Latency (ms)			
EO1 (p15)	15.1 (2.4)	14.8 (2.6)	14.9 (1.7)
EO1 (n21)	21.4 (2.5)	20.7 (2.2)	20.3 (2.1)
EO2 (p15)	14.9 (2.3)	14.8 (2.6)	14.6 (2.0)
EO2 (n21)	21.3 (2.6)	20.9 (2.2)	20.1 (1.8)
Cerebellar electrodes (CEP)			
Amplitude (μV)			
PO9 (p14/n20)	9.2 (9.4)	11.8 (12.3)	23.9 (15.6)
PO10 (p14/n20)	22.4 (14.8)	12.6 (9.7)	8.7 (10.3)
Latency (ms)			
PO9 (p14)	14.1 (15.0)	14.0 (2.2)	14.3 (2.1)
PO9 (n20)	19.9 (2.6)	20.0 (2.3)	19.6 (3.8)
PO10 (p14)	13.2 (2.2)	14.5 (2.5)	14.3 (2.2)
PO10 (n20)	20.7 (4.3)	20.6 (2.8)	20.5 (2.9)
SCM electrodes (cVEMP)			
SCM activation (μV)			
L SCM	24.5 (10.2)	29.3 (13.4)	57.2 (27.8)
R SCM	49.8 (21.9)	29.0 (14.5)	23.2 (8.7)
Raw amplitude (μV)			
L SCM (p16)	3.5 (5.1)	7.9 (10.1)	32.4 (28.3)
R SCM (p16)	27.5 (28.0)	6.4 (4.4)	3.9 (7.3)
Corrected ratio			
L SCM (p16)	0.1 (0.1)	0.2 (0.2)	0.5 (0.2)
R SCM (p16)	0.5 (0.4)	0.2 (0.2)	0.2 (0.3)
Latency (ms)			
L SCM (p16)	16.6 (1.0)	15.4 (1.5)	16.7 (1.4)
R SCM (p16)	16.0 (2.8)	15.7 (1.6)	16.0 (2.2)
TA electrodes			
Amplitude (% increase from baseline)			
L TA (SL)	21.8 (24.5)	30.0 (33.4)	16.9 (23.9)
R TA (SL)	32.1 (33.8)	27.9 (23.4)	23.6 (26.7)
Latency (ms)			
L TA (SL onset)	53.0 (5.7)	53.3 (3.6)	52.1 (3.6)
L TA (SL end)	82.7 (10.2)	84.9 (10.4)	83.3 (8.6)
R TA (SL onset)	53.4 (6.3)	53.1 (3.7)	51.1 (3.8)
R TA (SL end)	82.7 (9.8)	84.3 (10.1)	83.8 (8.4)

Mean (SD), *L*, left; *R*, right

**Table 5:** oVEMP, CEP, cVEMP and TA postural evoked responses across the differing head positions to Oz stimulation

	Head position		
	*L max*	*Neutral*	*R max*
Extraocular electrodes (oVEMP)			
Amplitude (μV)			
EO1 (n14/p19)	10.8 (7.4)	10.5 (6.6)	9.9 (6.0)
EO2 (n14/p19)	11.4 (6.8)	11.4 (7.3)	11.2 (5.8)
Latency (ms)			
EO1 (n14)	14.1 (2.5)	13.8 (1.7)	13.7 (1.2)
EO1 (p19)	19.1 (2.9)	19.0 (2.1)	19.2 (1.2)
EO2 (n14)	13.9 (2.5)	13.7 (1.7)	13.3 (1.4)
EO2 (p19)	19.6 (2.9)	18.8 (2.2)	18.8 (1.6)
Cerebellar electrodes (CEP)			
Amplitude (μV)			
PO9 (p15/n20)	24.0 (26.1)	17.9 (7.9)	73.2 (77.8)
PO10 (p15/n20)	74.0 (47.6)	15.7 (9.2)	20.9 (19.1)
Latency (ms)			
PO9 (p15)	14.8 (2.1)	15.4 (1.5)	15.0 (2.2)
PO9 (n20)	19.2 (1.8)	20.5 (1.3)	19.4 (2.3)
PO10 (p15)	13.9 (1.8)	15.2 (1.9)	14.7 (2.2)
PO10 (n20)	19.0 (1.8)	19.9 (1.8)	19.3 (2.9)
SCM electrodes (cVEMP)			
SCM activation (μV)			
L SCM	17.6 (6.8)	17.0 (8.0)	18.4 (6.8)
R SCM	28.3 (15.7)	16.9 (7.1)	35.3 (18.1)
Raw amplitude (μV)			
L SCM (p20)	5.8 (4.9)	4.0 (5.4)	28.7 (27.4)
R SCM (p20)	22.8 (27.2)	5.6 (4.7)	7.5 (6.2)
Corrected ratio			
L SCM (p20)	0.3 (0.2)	0.2 (0.2)	0.7 (0.4)
R SCM (p20)	0.6 (0.4)	0.3 (0.2)	0.4 (0.3)
Latency (ms)			
L SCM (p20)	20.2 (1.6)	20.1 (0.9)	19.8 (2.1)
R SCM (p20)	19.5 (2.2)	19.4 (1.0)	19.4 (2.0)
TA electrodes			
Amplitude (% increase from baseline)			
L TA (SL)	−5.4 (10.8)	−5.3 (10.1)	−4.0 (7.7)
R TA (SL)	−3.6 (11.7)	−4.4 (14.2)	−5.0 (9.3)
Latency (ms)			
L TA (SL onset)	51.0 (3.0)	53.6 (3.7)	54.4 (4.0)
L TA (SL end)	82.3 (4.3)	83.0 (2.6)	82.5 (3.4)
R TA (SL onset)	54.5 (2.4)	53.1 (3.8)	55.0 (4.0)
R TA (SL end)	81.8 (1.4)	82.2 (2.9)	82.9 (1.5)

Mean (SD), *L*, left; *R*, right

For the extraocular electrodes, the polarity of the response differed between the two midline stimulation sites. Nz stimulation produced initial positive-negative (p15-n21) peaks, whereas Oz stimulation produced initial negative-positive (n14-p19) peaks, consistent with previous observations [23]. The oVEMP amplitude was not modulated by head position (Nz stimulation: *F*_(1.5,14.6)_ = 1.2, *P* = 0.325; Oz stimulation: *F*_(1.1,11.3)_ = 0.2, *P* = 0.686). The latencies for the p15, n21, n14 and p19 oVEMP peaks were unaffected by head position (Nz stimulation: *F*_(1.3,13.6)_ = 0.2, *P* = 0.728; Oz stimulation: *F*_(1.4,14.8)_ = 2.6, *P* = 0.120).

For the cerebellar electrodes, the effects were similar to the mastoid stimulation, with the VsCEP amplitude increasing on the side opposite to the direction of head rotation for both midline stimulus sites (Nz stimulation: *F*_(1.3,12.7)_ = 14.7, *P* = 0.001; Oz stimulation: *F*_(1.2,11.9)_ = 12.4, *P* = 0.003). Unlike the oVEMP, both VsCEPs began with positivity despite the reversal of induced movement. VsCEP latencies (p14, p15 and n20 peaks) were unaffected by head position (Nz stimulation: *F*_(1.7,17.4)_ = 0.8, *P* = 0.459; Oz stimulation: *F*_(1.4,13.4)_ = 1.6, *P* = 0.234).

For the SCM electrodes, responses were dominated by the muscle stretch component (n2), especially for Nz stimulation, and we therefore focused mainly on the initial positive peak for the analyses (p16 for Nz stimulation and p20 for Oz stimulation). The mean latency of the n2 peak of the stretch reflex averaged across sides was 32.3 and 31.8 ms (max left), 33.0 and 31.3 ms (neutral) and 32.5 and 30.6 ms (max right) for Nz and Oz stimulation respectively. For the initial positive peak, the midline sites produced larger responses on the side opposite to the direction of head rotation, and this was for both raw (Nz stimulation: *F*_(1.0,10.4)_ = 10.3, *P* = 0.008; Oz stimulation: *F*_(1.0,10.6)_ = 7.0, *P* = 0.022) and corrected amplitudes (Nz stimulation: *F*_(1.3,13.4)_ = 19.1, *P* < 0.001; Oz stimulation: *F*_(1.7,17.2)_ = 12.7, *P* < 0.001). Latencies for the p16 and p20 peaks were not affected by head position (Nz stimulation: *F*_(1.7,17.6)_ = 0.8, *P* = 0.458; Oz stimulation: *F*_(1.7,17.6)_ = 0.1, *P* = 0.852).

Unlike the mastoid stimulation sites, lower limb responses in TA were not affected by head position (Nz stimulation: *F*_(1.6,16.0)_ = 1.4, *P* = 0.274; Oz stimulation: *F*_(1.5,14.6)_ = 0.2, *P* = 0.719) but the polarity of the SL postural response differed depending on the site of stimulation. Nz stimulation produced an initial SL excitation, while for Oz stimulation, the initial SL response was inhibitory. Latencies for the SL onset and SL end periods were unaffected by head position (Nz stimulation: *F*_(1.6,16.2)_ = 2.2, *P* = 0.152; Oz stimulation: *F*_(1.111.6)_ = 0.3, *P* = 0.618), and there was no difference in latencies between the limbs (Nz stimulation: *F*_(1,10)_ = 0.5, *P* = 0.509; Oz stimulation: *F*_(1,10)_ = 0.2, *P* = 0.668).

## Discussion

Cerebellar cortical output is solely from the Purkinje cells (PC) and is inhibitory to the cerebellar deep nuclei [24, 25]. The postural effects of cerebellar lesions vary depending upon location [26], and our recordings of VsCEPs and associated ECeG are likely to be mainly generated from within the posterior lobe of the cerebellum, given its proximity to the posterior fossa electrode locations. Source localisation has shown dominant cerebellar sources in lobules VIIIA/B bilaterally, probably in the intermediate or paravermal zone [16]. PCs from the paravermis project primarily via the interposed nuclei with extensive targets within the brainstem [27]. All three reflexes that we have examined are thought to be primarily mediated via the brainstem and are therefore potential targets of cerebellar outflow.

Neck muscle spindle afferents project to the central cervical nucleus (CCN) which has an excitatory monosynaptic projection to the contralateral lateral, descending and medial vestibular nuclei [10, 28]. Furthermore, the CCN has a powerful, predominantly contralateral mossy fibre projection to the cerebellum [29, 30]. The increase in tonic high-frequency activity in the ECeG that we recorded (Fig. 3) is thus likely to be a reflection of increased mossy fibre and hence parallel fibre input, resulting in an increase in simple spike activity with differing degrees of head rotation.

The horizontal head acceleration within the transmastoid plane is the physiological stimulus for the utricle [31]. Previous evidence has shown that the smoothed impulsive stimulus used here, when applied to the mastoid, has effects consistent with utricular activation [11, 13]. The predominant inputs for the stimuli applied here are likely also to be vestibular, and the VsCEP latencies are distinct from those for afferents arising from the neck [16]. There are four major vestibular nuclei: the superior, lateral (or Deiter’s), medial and descending and utricular fibres mainly terminate in the lateral nucleus as well as some projecting to the descending and medial nuclei [32]. For a positive applied impulse, accelerating the head to the opposite side, it is likely that the medial part of the ipsilateral utricle is most strongly activated, while stimulation at Nz or Oz will have the same effects on both utricles but with opposing responses from the two sites [20, 23, 31] as well as activating axial muscle receptors [33]. The vestibular apparatus has an important role in stabilising gaze and head and body posture. Vestibular projections are known to be distributed to the eyes, cerebellum, neck and legs [32]. Likewise, our impulsive stimulus has been shown to evoke short latency reflexes in all these targets [12–15, 20, 22]. A range of effects occurred in response to the head rotation for the different reflex pathways, consistent with their differing physiological roles. The importance of vestibular reflex effects for the eyes, for which head-on-body posture has no relevance, may explain why there is little effect of body posture on responses of vestibular nucleus neurones [34].

The oculomotor effects (oVEMPs) were distinct for the two eyes with initial contralateral n12 and ipsilateral n15 peaks [13]. For these reflexes, the effect of head rotation was minimal. The role of vestibulo-ocular reflexes is to stabilise gaze, so head position per se is largely irrelevant. The short-latency pathway underlying the oVEMP is predominantly crossed [35] and, under conventional recording conditions, is mainly generated by the inferior oblique muscle [36, 37]. Cellular recordings have largely concentrated on the projection to the medial rectus and abducens nucleus. Vestibular input to the contralateral medial rectus component of the oculomotor nucleus appears to have a prominent disynaptic component and to travel via the ipsilateral abducens nucleus and then the medial longitudinal fasciculus (MLF) or the nearby ascending tract of Deiters to the contralateral oculomotor nucleus [38, 39]. Uchino et al. [40] found monosynaptic and disynaptic projections from the utricular nerve to the ipsilateral abducens nucleus and evidence of disynaptic projections to the contralateral medial rectus via the oculomotor nucleus. A similar projection to the inferior oblique muscle, the main generator of the oVEMP, would not be affected by changes in activity in neck afferents and thus be the basis of our observations for the oVEMP. The constancy of the oVEMP responses confirms that the vestibular volley that we evoked did not itself change significantly with head position. Midline stimulation evoked opposite initial responses in the oVEMPs for the two sites of midline stimulation [23]. Importantly, this did not alter the initial polarity of the VsCEPs, which remained positive, consistent with their origin as CFRs [15].

As previously reported [14], VsCEPs were the largest over PO9 and PO10 (Table 1). These showed profound modulation by head orientation in yaw, with a 5–6-fold increase in amplitude with the maximum degree of rotation compared to neutral. This observation suggests that these potentials are best recorded with the head fully rotated. The olivary projection to the cerebellum is nearly exclusively crossed [41], and the latencies associated with the two directions of rotation were different. The shortest latency was recorded from over the contralateral cerebellar hemisphere when the head was rotated towards the side of the (positive) stimulus. The later VsCEP response appears to arise from the ipsilateral cerebellum evoked when the direction of acceleration reverses [14]. As noted above, evidence, including the post-response pausing in the high-frequency ECeG, suggests that the VsCEP is a CFR arising from the Purkinje neurones [15]. Given its latency, it is likely to be generated by a two-synapse projection, one at the level of the vestibular nucleus and the second at the level of the inferior olive, and thence to the contralateral cerebellum. The latency is too short for indirect paths such as via the sensory cortex [42]. The CCN projects via mossy fibres to the anterior lobe and posterior vermis, but also projects to the inferior olive and to the vestibular nuclei [43]. The modulation of the VsCEP by neck afferents could, therefore, occur at a number of levels, including within the cerebellar cortex via granule cells and parallel fibres, or within the brainstem at the level of the vestibular nucleus or the inferior olive. The increase in power noted for the cerebellar hemispheres with head rotation is consistent with increased tonic activity, possibly due to increased mossy fibre input, but the VsCEP responses did not simply follow the changes in the tonic activity.

The cVEMP was increased with head rotation. Increases in the cVEMP amplitude in parallel with the level of tonic activation are well described [44–46], and this explained most of the increase in the cVEMP occurring with head rotation. Nevertheless, after scaling for the level of activation, there was additional facilitation with the head rotated away from the stimulated mastoid. Given that the pathway underlying the cVEMP appears to consist of a disynaptic projection via the medial vestibulospinal tract [47], this finding suggests that the facilitation occurred at the level of the medial vestibular nucleus.

A cardinal feature of cerebellar disease is a change in postural responses (ataxia-[48]). The profound effect of head rotation in intact humans was first demonstrated for (galvanic) vestibular-evoked postural reflexes [1]. Direct recordings indicate frequent convergence of both vestibular and neck afferent input on the Purkinje cells [49], and neck afferents appear capable of altering vestibulospinal reflexes, an effect dependent upon the cerebellar vermis [50]. In human subjects, Kammermeier et al. [51] reported the loss of galvanic-evoked vestibular postural reflexes in cerebellar disease, and Lam et al. [52] showed reductions in vestibular postural reflexes with suppression of the vermis. In our study, the effects of head rotation were most clearly seen in postural reflexes. Unlike the other reflexes studied, both legs showed the same changes in excitability, consistent with them being mediated by a bilaterally-projecting pathway such as the reticulospinal tract [53]. Our subjects were asked to lean backwards to increase tonic activity and responses for TA [33]. The VsCEP changes suggest that the postural effects may be mainly mediated through the cerebellar hemisphere, opposite to the direction of head rotation (Fig. 5). While we cannot exclude changes in the gain of SL reflexes in TA being partly due to changes in tonic EMG activity, the reversal of the effects of lateralised vestibular stimulation with head rotation is well established [2, 12]. If it is assumed that the cerebellar outflow on each side mainly facilitates anterior trunk movement, the observed effects of head rotation on postural responses can be explained by the gated excitation we have shown. The modulation of VsCEPs also occurred with midline stimulation, but the postural effects were constant. It is likely that the effects on vestibular afferents on the two sides were similar and additive under these conditions.

Studies on the basis of classical conditioning have implicated a role for the cerebellum and for disinhibition of the Purkinje discharge in particular as the means by which the conditioned stimulus evokes the reflex response [54]. The SL components of our evoked postural responses had similar durations to those of the post-excitation inhibition of cerebellar activity shown by wavelet and RMS measures but preceded them with the ECeG inhibition beginning around 18 ms and the SL onset beginning at approximately 52 ms. Each lasted a little more than 30 ms. An effect of the period of inhibition would be consistent with the role suggested for cerebellar output on reticulospinal neurons proposed by Eccles et al. [55]. The post-CFR pausing may also be part of a broader role in facilitating short-latency motor responses to specific stimuli, including the otolith-evoked blink reflex [22]. A corollary of this suggestion would be that reflexes with latencies less than 20 ms cannot be influenced by this means of cerebellar disinhibition, and, as a consequence, it would not be possible to condition such reflexes through a cerebellar mechanism. This would apply to the earliest part of the oVEMP as well as to other short-latency reflexes, such as the R1 component of the blink reflex. This consideration applies primarily to associative learning, but other sites and mechanisms of cerebellar plasticity may not have this limitation [56].

## Conclusion

The effect of head rotation on the reflexes evoked by mastoid stimulation differed for different targets, consistent with their physiological roles. Where head position was largely irrelevant, i.e. for the oVEMP, the changes with different head postures were minimal. For the cVEMP, an effect was present but less powerful than the changes in the level of tonic activation of the muscle itself. Our findings confirm the profound effect of head rotation on postural reflexes evoked by lateralised vestibular stimulation when standing and show associated changes in the VsCEPs, tonic ECeG and the post-CFR pause in the ECeG. Our study has shown systematic variations in the amplitude of VsCEPs evoked by lateral vestibular afferent stimulation, effects which may in turn be transmitted to brainstem targets. This as well as the changes in overall cerebellar activity may be a means of controlling the gain of brainstem reflexes. Local reflex gain control also occurs at the segmental level through the degree of tonic contraction [57]. A cerebellar projection onto reticulospinal neurones is a possible mechanism for the modulation of postural reflexes [58, 59].
